# Electrophilic Bromination in Flow: A Safe and Sustainable Alternative to the Use of Molecular Bromine in Batch

**DOI:** 10.3390/molecules24112116

**Published:** 2019-06-04

**Authors:** Reinout Van Kerrebroeck, Pieter Naert, Thomas S. A. Heugebaert, Matthias D’hooghe, Christian V. Stevens

**Affiliations:** SynBioC research Group, Department of Green Chemistry and Technology, Ghent University, Coupure Links 653, 9000 Ghent, Belgium; Reinout.vankerrebroeck@ugent.be (R.V.K.); Pieter.Naert@ugent.be (P.N.); Thomas.heugebaert@ugent.be (T.S.A.H.); Matthias.Dhooghe@UGent.be (M.D.)

**Keywords:** aromatic substitution, bromination, C–H activation, ionic liquids, microreactors

## Abstract

Bromination reactions are crucial in today’s chemical industry since the versatility of the formed organobromides makes them suitable building blocks for numerous syntheses. However, the use of the toxic and highly reactive molecular bromine (Br_2_) makes these brominations very challenging and hazardous. We describe here a safe and straightforward protocol for bromination in continuous flow. The hazardous Br_2_ or KOBr is generated in situ by reacting an oxidant (NaOCl) with HBr or KBr, respectively, which is directly coupled to the bromination reaction and a quench of residual bromine. This protocol was demonstrated by polybrominating both alkenes and aromatic substrates in a wide variety of solvents, with yields ranging from 78% to 99%. The protocol can easily be adapted for the bromination of other substrates in an academic and industrial environment.

## 1. Introduction

One of the major challenges in synthetic chemistry concerns the transformation of a relatively inert C–H bond into a more active functional group. This can be done with transition metal catalysis in the so-called C–H activation [1]. An alternative option is direct bromination with molecular bromine (Br_2_), forming an organobromide. This is an optimal first step for most synthetic pathways since the high reactivity of this formed organobromide allows for an easy subsequent conversion into a wide variety of other functional groups [2,3,4]. This reaction is, however, plagued by the use of toxic and corrosive reagents [5] and a high risk of runaway reactions [6].

To circumvent these problems, brominations are often performed with alternative bromination reagents, most commonly organic molecules containing a Br(I)-species. This bromine(I) can be bonded to nitrogen (e.g., N-bromosuccinimide or tribromoisocyanuric acid [7,8,9]); iodine(III) [10]; carbon (e.g., CBr_4_ [11]), or even three bromine atoms bonded together in Br_3_^−^, countered by an organic cation, possibly forming an ionic liquid [12,13]. Although these alternatives provide a number of benefits, especially for academic laboratories, they still are far from perfect—they are expensive, are often still toxic and unsustainable, produce organic by-products, and most of them use molecular bromine in their synthesis, thus not solving but only shifting the problem [9,14,15].

Another option is to use Br_2_, but to minimize the hazards by producing it in situ, by reacting reduced bromide (in the form of hydrobromic acid or a bromide salt) with an oxidizing agent, e.g., hydrogen peroxide and other peroxides [16,17,18], Oxone [19], bromate [20,21], or even air or oxygen at elevated temperatures or with a catalyst [22]. This approach solves the storage and transport problem, but the risk of a runaway reaction is still present. Selectivity can also be problematic due to the highly reactive nature of the used reagents [23].

All these problems can be avoided by the development of a continuous flow process. The high surface over volume ratio greatly diminishes the risk of a runaway reaction and ensures a good parameter control, meaning optimal selectivities and conversions can be achieved with minimal reagent or solvent [24]. This high selectivity and efficiency of continuous flow bromination has been demonstrated in previous works [15,25,26,27,28]. Additionally, by generating the bromine in situ, as has been done in batch, the hazards associated with transport and storage can be circumvented, and the production rate can be tailored to the consumption rate. In situ generation of Br_2_ from HBr and H_2_O_2_ resulted in a safe and efficient bromination protocol [29], and if the residual bromine is quenched before leaving the reactor, the risk of accidental release of bromine drops to practically zero, since at any given time there is not enough Br_2_ present in the reactor to harm either the environment or the people performing the reaction in case of an accidental spill.

However, a comprehensive study of the versatility of this in situ-generated bromine in flow, especially for electrophilic brominations, has not yet been performed. This paper provides a broadly applicable protocol for electrophilic brominations, using in situ-generated bromine from bromide and an oxidant.

## 2. Results and Discussion

**Figure 1:**
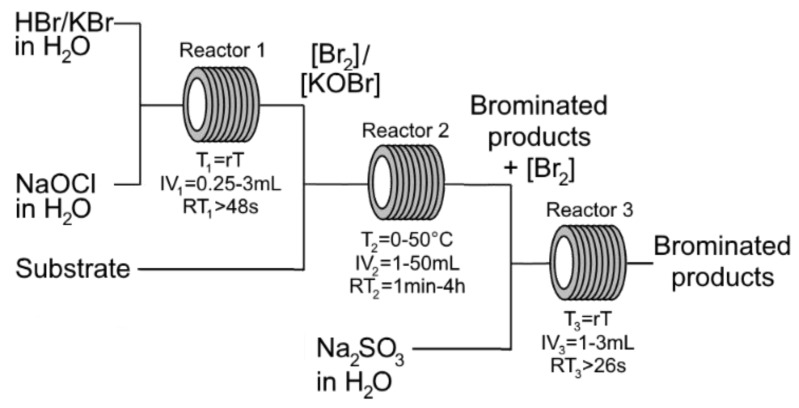
A general scheme for the bromination of organic compounds in flow. The exact parameters used per substrate can be found in Table 1 and Section 3 Materials and Methods.

The protocol was devised with the main goal of reducing the risk of bromine exposure. This was achieved by generating the bromine in situ (reactor 1), performing the reaction (reactor 2), and quenching the unreacted bromine before it leaves the reactor (reactor 3), as depicted in Figure 1.

Preliminary experiments demonstrated that a combination of the first two steps (oxidation and bromination) is not possible because of side product formation by oxidation and chlorination. A number of oxidizing agents were considered: oxygen, hydrogen peroxide, sodium hypochlorite, and Oxone. Sodium hypochlorite was selected because it is cheap, usable at any given pH, and does not need elevated temperatures or metal catalysts. A minimal residence time of 48 s at room temperature was found to be sufficient to complete the bromine oxidation in acidic environment (i.e., starting from HBr), while in an alkaline environment (i.e., starting from KBr), the reaction proceeded more slowly, although 5 min at room temperature was also sufficient:

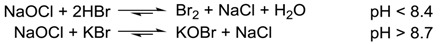
(1)

Br_2_ and KOBr are brominating agents operating via the same mode of action. Both reagents underwent a nucleophilic substitution on the bromine, expelling, respectively, a bromide group, a very good leaving group, or potassium oxide (KO^−^), a very poor leaving group. This raises the idea that Br_2_ will be a stronger brominating agent than KOBr. The bromination of (*E,E,Z*)-1,5,9-cyclododecatriene, which went smoothly in the acidic medium (using Br_2_) but did not occur at all in alkaline medium (using KOBr), confirmed this hypothesis. Hence, bromination is strongly favourable in acidic media, since both the kinetics and conversions are substantially higher. After bromination, the residual bromine was quenched with Na_2_SO_3_, as this is cheap, water soluble, and does not produce any solid by-products. A residence time of two minutes at room temperature was found to be sufficient to quench all of the unreacted bromine, using two equivalents of Na_2_SO_3_ per equivalent of formed bromine. In conclusion, this approach (I) generates bromine in situ directly coupled to its consumption, (II) eliminates exposure to bromine outside the reactor, and (III) avoids intensive downstream purification (i.e., flash chromatography) due to the absence of organic side products caused by the use of purely inorganic reagents in the bromination.

The protocol was used to polybrominate different industrially relevant substrates as a proof of concept. 2,4,6-Tribromophenol (Table 1, entry 1) is a biocide in wood and an intermediate in the production of flame retardants [30]. 2,2′,6,6′-Tetrabromobisphenol A (Table 1, entry 2) and 1,2,5,6,9,10-hexabromocyclododecane (Table 1, entry 3) are important flame retardants, used, respectively, in polycarbonate and epoxy resins and polystyrene [31].

Even though these retardants are less bio-persistent than other brominated flame retardants, there is still cause for concern [32]. However, their production is necessary, since flame retardants are very much needed for fire safety, and alternative flame retardants also cope with toxicity and bio-persistence issues [32]. It is thus of vital importance that their synthesis is performed as efficiently as possible, with maximal conversion and yield. Potential organic side products, through partial bromination, are also bio-persistent organobromides and have to be avoided. To achieve this goal, an excess of brominating agent was used to ensure full conversion and prevent organic side product formation. This is both economically and environmentally viable due to the low cost and green nature of the used brominating agent and its by-products (inorganic salts) compared to the organic substrate. With this approach, full conversions and good to excellent yields (83–97%) were achieved. Solvent choice was made purely based on solubility, since the solvent polarity had only a small effect on the reaction rate. None of the tested solvents were found to be more prone to bromination than the substrates. A suitable organic solvent for the bromination of cyclododecatriene (Table 1, entry 3) was found through a screening of the common solvents and their mixtures by COSMOquick [33,34,35]. Since phase transfer occurs quickly in microreactors and Br_2_ dissolves preferentially in an apolar solvent, mixing was not a rate limiting factor in the obtained two-phase system [36]. Another big advantage of reaching full conversion, mainly from an industrial point of view, is that thanks to the absence of organic side or by-products, there was no need for any chromatographic purification. Simple extraction(s) led to the pure end products.

Bromothymol blue is a pH indicator, and eosin Y (Table 1, entry 4) is mainly used as a histology stain, for example in the H&E (Hematoxylin and Eosin) stain. Both, however, suffer from a poor solubility in acidic water or organic solvents. (Bromo)thymol blue dissolves well in a homogeneous alkaline water/ethanol mixture, and it was attempted to perform the reaction in a one-phase system. Even though the inherently lower reactivity of KOBr compared to Br_2_ causes the need for longer reaction times (4 h), the reaction seemed to work with 100% conversion. However, due to problems with the work-up, it was not possible to confirm this by obtaining pure bromothymol blue, and caution has to be taken in saying that switching from Br_2_ to KOBr is a possible solution to solve solubility problems. It has been proven in literature, however, that KOBr is a valuable brominating agent, for example, in cases where an alkaline environment is necessary to activate the substrate by deprotonating the position that has to be brominated, in this case a methylsulfon(ate) [25].

**Scheme 1:**
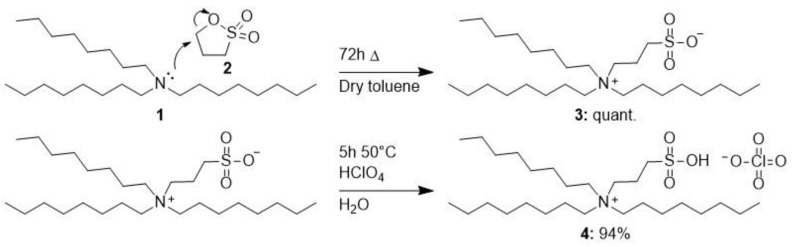
The synthesis of the ionic liquid, trioctyl-(3-sulfopropyl)ammonium perchlorate **4**.

While eosin Y could also be dissolved in alkaline water, the higher oxidation potential of KOBr compared to Br_2_ caused the substrate to be degraded rather than brominated. The oxidation potential of Br_2_ was low enough not to degrade eosin Y, so the bromination had to take place in an acidic environment. Since a suitable water-immiscible solvent was not found, even when performing screenings with COSMOquick [33,34,35], the attention was turned to ionic liquids. Most ionic liquids either did not dissolve fluorescein properly or were water-miscible. Trioctyl-(3-sulfopropyl)ammonium perchlorate **4**, however, was found to be a suitable solvent. Trioctyl-(3-sulfopropyl)ammonium perchlorate **4** is not commercially available so it was synthesized according to Scheme 1, based on the literature protocol for the synthesis of trioctyl-(3-sulfopropyl)ammonium bistriflimide [37]. Perchlorate was chosen as anion, which is cheap, oxidative stable, and resistant to ion exchange [38]. This synthetic route involved N-alkylation of tertiary trioctylamine **1** with 1,3-propane sultone **2** to furnish the zwitterionic sulfonate **3**. Treatment of the latter with perchloric acid finally provided the desired ionic liquid **4.** A 1/9 mixture of this trioctyl-(3-sulfopropyl)ammonium perchlorate **4** and 2-methyltetrahydrofuran dissolved fluorescein and had an acceptable viscosity. This proves that the presented protocol is not only viable in a wide range of conventional solvents, ranging from the most bio-persistent, e.g., chlorinated solvents, to the most sustainable, e.g., 2-methyltetrahydrofuran, but also in ionic liquids.

## 3. Materials and Methods

### 3.1. General Procedure

The reactor was constructed according to the scheme presented in Figure 1. All connections were poly(ethylene-co-tetrafluoroethylene) (ETFE) T-mixers and Luer lock adapters, as polyether ether ketone (PEEK) gets easily degraded by Br_2_. All tubing was polytetrafluoroethylene (PTFE) and had, unless stated otherwise, an internal diameter (ID) of 1 mm.

Hydrobromic acid was bought in a 48 w% solution in water from Acros Organics, and sodium hypochlorite in a 13% active chlorine solution in water from Fisher Scientific (Hampton, NH, USA), but both were titrated (acid-base and iodometric), since the exact concentration can differ from the labeled one, especially for NaOCl. This concentration also diminishes over time, even when refrigerated. Anhydrous Na_2_SO_3_ was bought from Fisher Scientific and a stock solution was made in water (200 g/L).

### 3.2. 2,4,6-Tribromophenol (Table 1, Entry 1)

Phenol was purchased from Acros Organics and used without any purification. The reactor was built as depicted in Figure 1. Both reactor 1 and 3 had an ID of 1 mm and a volume of 3 mL, but reactor 2 had an ID of 2.4 mm and a volume of 50 mL. Reactor 2 was placed in a 50 °C water bath. The HBr and NaOCl were titrated to find a concentration of 8.89 M for HBr and 1.45 M for NaOCl. A phenol solution of 0.194 M in CHCl_3_ was made, as well as a solution of 200 g/L Na_2_SO_3_ in H_2_O. The latter three solutions were pumped by the peristaltic pumps of the Vapourtec E-series (VapourTec Ltd, Bury Saint Edmunds, UK), while the HBr was loaded in a syringe and placed on a Chemyx Fusion 100 syringe pump (Chemyx Inc, Stafford, TX, USA). The flow rates were 0.196 mL/min for HBr (9 eq.), 0.804 mL/min for NaOCl (6 eq.), 1 mL/min for phenol (1 eq.), and 1.468 mL/min for Na_2_SO_3_ (12 eq.). This resulted in a residence time of 25 min in reactor 2, the bromination reactor. After running the reaction for three complete residence times, the outlet was collected for 30 min.

The two phases were separated, and the water phase was extracted with 3 × 50 mL CHCl_3_. All organic phases were combined, dried with MgSO_4_, filtrated, and evaporated in vacuo. Drying under a high vacuum yielded 1.83 g of pure 2,4,6-tribromophenol (yield = 95%). Purity was confirmed by liquid chromatography-mass spectrometry (LC–MS) (Agilent Technologies, Santa Clara, CA, USA) and nuclear magnetic resonance spectroscopy (NMR), both ^1^H-NMR and ^13^C-NMR (Bruker, Billerica, MA, USA). These spectra can be found in the Supporting Information.

### 3.3. 2,2′,6,6′-Tetrabromobisphenol A (Table 1, Entry 2)

Bisphenol A was purchased from Sigma-Aldrich (St. Louis, MO, USA) and used without any purification. The reactor was built as depicted in Figure 1. All tubing had an internal diameter of 1 mm, and the three reactors had volumes of 2, 10, and 3 mL respectively. The HBr and NaOCl were titrated to find a concentration of 8.89 M for HBr and 1.45 M for NaOCl. A bisphenol A solution of 0.233 M in Et_2_O was made, as well as a solution of 200 g/L Na_2_SO_3_ in H_2_O. The latter three solutions were pumped by the peristaltic pumps of the Vapourtec E-series, while the HBr was loaded in a syringe and placed on a Chemyx Fusion 100 syringe pump. The flow rates were 0.393 mL/min for HBr (7.5 eq.), 1.61 mL/min for NaOCl (5 eq.), 2 mL/min for bisphenol A (1 eq.), and 2.94 mL/min for Na_2_SO_3_ (10 eq.). This resulted in a residence time of 2 min and 30 s in reactor 2, the bromination reactor. After running the reaction for three complete residence times, the outlet was collected for 10 min.

The two phases were separated, and the water phase was extracted with 3 × 50 mL Et_2_O. All organic phases were combined, dried with MgSO_4_, filtrated, and evaporated in vacuo. Drying under a high vacuum yielded 2.10 g of pure 2,2′,6,6′-tetrabromobisphenol A (yield = 83%). A perfect selectivity was obtained, while incorrect parameters resulted in the degradation of the carbon skeleton and production of tribromophenol (δ = 7.64). Purity was confirmed by LC–MS, ^1^H-NMR.and ^13^C-NMR. These spectra can be found in the Supporting Information.

### 3.4. 1,2,5,6,9,10-Hexabromocyclododecane (Table 1, Entry 3)

(*E,E,Z*)-1,5,9-cyclododecatriene was bought from Fisher Scientific and used without any purification. The reactor was built as depicted in Figure 1. All tubing had an internal diameter of 1 mm, and the three reactors had volumes of 0.4, 1, and 1 mL respectively. Reactor 2 was cooled by an ice/water bath to prevent overbromination forming hepta- and octabromocyclododecane. The HBr and NaOCl were titrated to find a concentration of 9.16 M for HBr and 1.31 M for NaOCl. An (*E,E,Z*)-1,5,9-cyclododecatriene solution of 0.107 M in a 1/2 mixture of cyclohexane and dichloromethane was made, as well as a solution of 200 g/L Na_2_SO_3_ in H_2_O. All reagents were loaded in syringes, but while the HBr, NaOCl and Na_2_SO_3_ were pumped by Chemyx Fusion 100 syringe pumps, the cyclododecatriene was pumped by a Harvard PhD 22/2000 pump. The flow rates were 0.132 mL/min for HBr (22.5 eq.), 1.313 mL/min for NaOCl (9 eq.), 0.5 mL/min for cyclododecatriene (1 eq.) and 0.609 mL/min for Na_2_SO_3_ (18 eq.). This resulted in a residence time of 1 min in reactor 2, the bromination reactor. After running the reaction for three complete residence times, the outlet was collected for 15 min.

The two phases were separated, and the organic phase was dried with MgSO_4_, filtrated and evaporated in vacuo. Drying under a high vacuum yielded 502 mg of pure 1,2,5,6,9,10-hexabromocyclododecane (yield = 97%). Purity was confirmed by LC–MS. This spectrum can be found in the Supporting Information.

### 3.5. Eosin Y (Table 1, entry 4)

Fluorescein was purchased from Acros Organics and used without purification. The reactor was built as depicted in Figure 1. All tubing had an internal diameter of 1 mm, and the three reactors had volumes of 0.4, 7, and 1 mL respectively. The HBr and NaOCl were titrated to find a concentration of 9.16 M for HBr and 1.29 M for NaOCl. A solution of 200 g/L Na_2_SO_3_ in H_2_O was made. Fluorescein (704.6 mg) was dissolved in 40 mL of a 1/9 volumetric mixture of trioctyl-(3-sulfopropyl)ammonium perchlorate and 2-methyltetrahydrofuran. A few drops of hydrobromic acid were added to improve the dissolution. This mixture was stirred until properly dissolved, exemplified by a clear deep red color. All reagents were loaded in syringes, but while the HBr, NaOCl and Na_2_SO_3_ were pumped by Chemyx Fusion 100 syringe pumps, the fluorescein was pumped by a Harvard PhD 22/2000 pump. The flow rates were 0.00338 mL/min for HBr (22.5 eq.), 0.00959 mL/min for NaOCl (9 eq.), 0.0259 mL/min for fluorescein (1 eq.), and 0.01559 mL/min for Na_2_SO_3_ (18 eq.). This resulted in a residence time of 3 h in reactor 2, the bromination reactor. After running the reaction for three complete residence times, the outlet was collected for 15 h.

The two phases were separated, and the water phase was discarded. A 50 mL value of isobutanol was added to the organic phase, and concentrated NaOH was added until the color changed from orange to dark red with a green shade. This mixture was extracted with 3 × 50 mL of water, during which the pH was held high enough to keep the color from changing back to orange. Since the end product is brightly coloured, this extraction can easily be followed up. It was noted that it was impossible to perform the extraction with full efficiency, resulting in a lower yield than expected. The water phases were combined, extracted once with 50 mL of petroleum ether to remove the residual organic solvents or ionic liquid, cooled to 0 °C and carefully acidified with concentrated H_2_SO_4_. The precipitated eosin Y was filtered off as an orange paste and was dried under a high vacuum. An 612.2 mg amount of pure eosin Y (yield = 78%) was obtained as an orange powder. Purity was checked by LC–MS, ^1^H-NMR.and ^13^C-NMR. These spectra can be found in the Supporting Information.

### 3.6. Synthesis of Trioctyl-(3-sulfopropyl)ammonium Perchlorate ***4***

**Figure 2:**
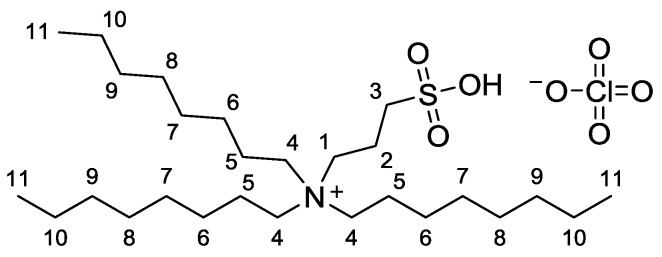
Trioctyl-(3-sulfopropyl)ammonium perchlorate.

In a 250 mL flame-dried flask the following were loaded: 1,3-propane sultone **2** (0.20 mol, 24.4 g), trioctylamine **1** (0.15 mol, 53.1 g), dry toluene (100 mL), and a stirring bar. The reaction mixture was heated under reflux under an inert Ar-atmosphere. After 72 h, the solvent was evaporated in vacuo, and the residual brown waxy residue was thoroughly washed with Et_2_O until white and dried under a high vacuum. The zwitterion **3** (Scheme 1) was obtained as a powder in quantitative yield, its purity was confirmed by ^1^H-NMR. 50% HClO_4_ in H_2_O was added (22.5 mol, 45.2 g), and the mixture was heated to 50 °C for 5 h, after which two phases had formed. A 50 mL amount of deionized water was added, and the mixture was extracted with 3 × 50 mL Et_2_O. Every piece of glassware that came into contact with the ionic liquid was rinsed with Et_2_O to minimize the losses and maximize the yield of the viscous ionic liquid. The combined organic phases were evaporated in vacuo and dried under a high vacuum until 81.32 g (94.1% overall yield). Pure trioctyl-(3-sulfopropyl)ammonium perchlorate **4** (Figure 2) was obtained as a very viscous brown liquid, and its purity was confirmed by ^1^H-NMR and ^13^C-NMR. These spectra can be found in the Supporting Information.

^1^H-NMR (400 MHz, acetone-*d*_6_): δ = 0.84 (t, *J* = 7 Hz, 9H; C^11^H_3_), 1.25–1.35 (m, 30H; C^6-10^H_2_), 1.77 (br. s, 6H, C^5^H_2_), 2.22 (br. s, 2H, C^2^H_2_), 3.14 (t, *J* = 7 Hz, 2H; C^3^H_2_, 3.58 (br. t, *J* = 8 Hz, 6H; C^4^H_2_), 3.35 (m, 2H; C^1^H_2_), 10.26 (br. s, D_2_O-exch. SO_3_H); ^13^C-NMR (100 MHz, acetone-*d*_6_), 13.7 (C^11^), 17.6 (C^2^), 21.5 (C^5^), 22.4 (C^10^), 26.0 (C^6^), 28.7 (C^7^), 28.9 (C^8^), 31.6 (C^9^), 47.9 (C^3^), 56.7 (C^1^), 58.7 (C^4^).

## 4. Conclusions

A safe and sustainable protocol for the generation, in situ use, and subsequent quench of bromine was described. The versatility of this protocol was shown by the polybromination of a variety of substrates in different solvents and even ionic liquids. By performing the reaction in flow, an inherently difficult and dangerous reaction was made safe and straightforward. The high conversion, easy work-up, and minimal waste generation due to the superior reaction parameter control in the microreactor are especially important in reactions like brominations, since potential organobromide side products are problematic due to their bio-persistence. By avoiding these, a very unsustainable, but nevertheless very important and necessary, reaction can be made considerably greener and safer.

## Figures and Tables

**Table 1:** Synthesis of four indicative organobromine compounds via in-situ Br_2_ generation and bromination.

Entry	Substrate	Product	Solvent	Eq. NaOCl	Eq. Br^−^	RT_2_	T_2_	Conversion	Yield
1	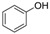	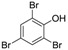	CHCl_3_	6	9	25 min	50 °C	100%	95%
2	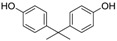	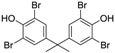	Et_2_O	5	7.5	150 s	rT	100%	83%
3	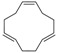	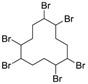	Cyclohexane/DCM	9	22.5	1 min	0 °C	100%	97%
4	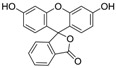	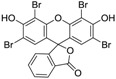	2-MeTHF/**4**	9	22.5	3 h	rT	100%	78%

## Supplementary Materials

The following are available online at https://www.mdpi.com/1420-3049/24/11/2116/s1: Spectra of all synthesized compounds.

## References

[1] Labinger J.A., Bercaw J.E. (2002). Understanding and exploiting C–H bond activation. Nature.

[2] Miyaura N., Yamada K., Suzuki A. (1979). A new stereospecific cross-coupling by the palladium-catalyzed reaction of 1-alkenylboranes with 1-alkenyl or 1-alkynyl halides. Tetrahedron Lett..

[3] Heck R.F., Nolley J.P. (1972). Palladium-catalyzed vinylic hydrogen substitution reactions with aryl, benzyl, and styryl halides. J. Org. Chem..

[4] King A.O., Okukado N., Negishi E.-i. (1977). Highly general stereo-, regio-, and chemo-selective synthesis of terminal and internal conjugated enynes by the Pd-catalysed reaction of alkynylzinc reagents with alkenyl halides. J. Chem. Soc. Chem. Comm..

[5] NIOSH Pocket Guide to Chemical Hazards: Bromine. https://www.cdc.gov/niosh/npg/npgd0064.html.

[6] Urben P. (2017). Bretherick’s Handbook of Reactive Chemical Hazards.

[7] Ng W.H., Shing T.K., Yeung Y.-Y. (2018). Mild and Efficient Vicinal Dibromination of Olefins Mediated by Aqueous Ammonium Fluoride. Synlett.

[8] De Almeida L.S., Esteves P.M., de Mattos M.C. (2006). Tribromoisocyanuric acid: A new reagent for regioselective cobromination of alkenes. Synlett.

[9] Kitching M.O., Dixon O.E., Baumann M., Baxendale I.R. (2017). Flow-Assisted Synthesis: A Key Fragment of SR 142948A. Eur. J. Org. Chem..

[10] Satkar Y., Ramadoss V., Nahide P.D., García-Medina E., Juárez-Ornelas K.A., Alonso-Castro A.J., Chávez-Rivera R., Jiménez-Halla J.O.C., Solorio-Alvarado C.R. (2018). Practical, mild and efficient electrophilic bromination of phenols by a new I (iii)-based reagent: The PIDA–AlBr 3 system. RSC Adv..

[11] Khusnutdinov R., Shchadneva N., Mayakova Y.Y., Yulamanova A., Khazipova A., Kutepov B. (2018). Halogenation of Diamantane by Halomethanes Under the Action of Zeolites. Russ. J. Gen. Chem..

[12] Chaudhuri M.K., Khan A.T., Patel B.K., Dey D., Kharmawophlang W., Lakshmiprabha T., Mandal G.C. (1998). An environmentally benign synthesis of organic ammonium tribromides (OATB) and bromination of selected organic substrates by tetrabutylammonium tribromide (TBATB). Tetrahedron Lett..

[13] Kaushik M., Polshettiwar V. (2006). N-octylquinolinium tribromide: A task specific quinoline based ionic liquid as a new brominating agent. Indian. J. Chem..

[14] Attanasi O.A., Berretta S., Favi G., Filippone P., Mele G., Moscatelli G., Saladino R. (2006). Tetrabromo hydrogenated cardanol: Efficient and renewable brominating agent. Org. Lett..

[15] Zhao Y., Li Z., Yang C., Lin R., Xia W. (2014). Visible-light photoredox catalysis enabled bromination of phenols and alkenes. Beilstein J. Org. Chem..

[16] Barhate N.B., Gajare A.S., Wakharkar R.D., Bedekar A.V. (1999). Simple and practical halogenation of arenes, alkenes and alkynes with hydrohalic acid/H2O2 (or TBHP). Tetrahedron.

[17] Podgoršek A., Stavber S., Zupan M., Iskra J. (2009). Environmentally benign electrophilic and radical bromination ‘on water’: H2O2–HBr system versus N-bromosuccinimide. Tetrahedron.

[18] Semwal R., Ravi C., Kumar R., Meena R., Adimurthy S. (2019). Sodium Salts (NaI/NaBr/NaCl) for the Halogenation of Imidazo-Fused Heterocycles. J. Org. Chem..

[19] Olsen K.L., Jensen M.R., MacKay J.A. (2017). A mild halogenation of pyrazoles using sodium halide salts and Oxone. Tetrahedron Lett..

[20] Adimurthy S., Ghosh S., Patoliya P.U., Ramachandraiah G., Agrawal M., Gandhi M.R., Upadhyay S.C., Ghosh P.K., Ranu B.C. (2008). An alternative method for the regio-and stereoselective bromination of alkenes, alkynes, toluene derivatives and ketones using a bromide/bromate couple. Green. Chem..

[21] Adimurthy S., Ramachandraiah G., Bedekar A.V., Ghosh S., Ranu B.C., Ghosh P.K. (2006). Eco-friendly and versatile brominating reagent prepared from a liquid bromine precursor. Green. Chem..

[22] Ghorpade A.K., Huddar S.N., Akamanchi K.G. (2016). Aq HBr–NaNO2–KI/air: A new catalytic system for α-monobromination of ketones. Tetrahedron Lett..

[23] de la Vega F., Sasson Y., Huddersman K. (1991). Selectivity in the liquid-phase bromination of aromatics catalyzed by zeolites. Zeolites.

[24] Movsisyan M., Delbeke E., Berton J., Battilocchio C., Ley S., Stevens C. (2016). Taming hazardous chemistry by continuous flow technology. Chem. Soc. Rev..

[25] Van Waes F.E., Seghers S., Dermaut W., Cappuyns B., Stevens C.V. (2014). Efficient continuous-flow bromination of methylsulfones and methanesulfonates and continuous synthesis of hypobromite. J. Flow. Chem..

[26] Cantillo D., Kappe C.O. (2017). Halogenation of organic compounds using continuous flow and microreactor technology. React. Chem. Eng..

[27] Battilocchio C., Bosica F., Rowe S.M., Abreu B.L., Godineau E., Lehmann M., Ley S.V. (2017). Continuous Preparation and Use of Dibromoformaldoxime as a Reactive Intermediate for the Synthesis of 3-Bromoisoxazolines. Org. Process. Res. Dev..

[28] Godineau E., Battilocchio C., Lehmann M., Ley S.V., Labes R., Birnoschi L., Subramanian S., Prasanna C.S., Gorde A., Kalbagh M. (2018). A Convergent Continuous Multistep Process for the Preparation of C4-Oxime-Substituted Thiazoles. Org. Process. Res. Dev..

[29] Yu W.-b., Yu D.-p., Zheng M.-m., Shan S.-t., Li Y.-j., Gao J.-r. (2012). Catalyst and solvent-free bromination of toluene derivatives by HBr-H2O2 with visible-light photocatalysis using a continuous-flow micro reactor. J. Chem. Res..

[30] Howe P., Dobson S., Malcolm H. (2005). 2, 4, 6-Tribromophenol and Other Simple Brominated Phenols.

[31] Alaee M., Arias P., Sjödin A., Bergman Å. (2003). An overview of commercially used brominated flame retardants, their applications, their use patterns in different countries/regions and possible modes of release. Environ. Int..

[32] Ali N., Dirtu A.C., Eede N.V.d., Goosey E., Harrad S., Neels H., Mannetje A., Coakley J., Douwes J., Covaci A. (2012). Occurrence of alternative flame retardants in indoor dust from New Zealand: Indoor sources and human exposure assessment. Chemosphere.

[33] Loschen C., Hellweg A., Klamt A. (2018). COSMOquick.

[34] Hornig M., Klamt A. (2005). COSMOfrag: A Novel Tool for High-Throughput ADME Property Prediction and Similarity Screening Based on Quantum Chemistry. J. Chem. Inf. Model..

[35] Loschen C., Klamt A. (2012). COSMOquick: A novel interface for fast σ-profile composition and its application to COSMO-RS solvent screening using multiple reference solvents. Ind. Eng. Chem. Res..

[36] Ahmed B., Barrow D., Wirth T. (2006). Enhancement of reaction rates by segmented fluid flow in capillary scale reactors. Adv. Synth. Catal..

[37] Dupont D., Raiguel S., Binnemans K. (2015). Sulfonic acid functionalized ionic liquids for dissolution of metal oxides and solvent extraction of metal ions. Chem. Commun..

[38] Naert P., Rabaey K., Stevens C.V. (2018). Ionic liquid ion exchange: Exclusion from strong interactions condemns cations to the most weakly interacting anions and dictates reaction equilibrium. Green Chem..

