# Supplementary material for: Electrophilic Bromination in Flow: A Safe and Sustainable Alternative to the Use of Molecular Bromine in Batch
Source: Molecules. 2019 Jun 4;24(11):2116. doi: 10.3390/molecules24112116 (PMC6600453; doi:10.3390/molecules24112116)
Supplement: Supplementary file 1 [file molecules-24-02116-s001.pdf]

molecules

MDPI

Supporting info

Electrophilic Bromination in Flow: a Safe and Sustainable Alternative to the Use of Molecular Bromine in Batch: Supporting Info

Reinout Van Kerrebroeck [1], Pieter Naert [1], Thomas S.A. Heugebaert [1], Matthias D'hooghe [1] and Christian V. Stevens [1,*]

[1] SynBioC research Group, Department of Green Chemistry and Technology, Ghent University, Coupure Links 653, 9000 Ghent, Belgium

* Correspondence: Chris.Stevens@UGent.be

1. Trioctyl-(3-sulfopropyl)ammonium perchlorate 4

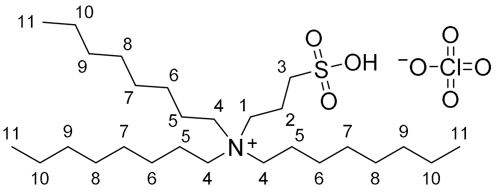

^{1}H NMR (400 MHz, acetone-d_6): δ=0.84 (t, J=7Hz, 9H; $C^{11}\underline{H}_3$), 1.25-1.35 (m, 30H; $C^{6\text{-}10}\underline{H}_2$), 1.77 (br. s, 6H, $C^5\underline{H}_2$), 2.22 (br. s, 2H, $C^2\underline{H}_2$), 3.14 (t, J=7Hz, 2H; $C^3\underline{H}_2$, 3.58 (br. t, J=8Hz, 6H; $C^4\underline{H}_2$), 3.35 (m, 2H; $C^1\underline{H}_2$), 10.26 (br. s, D_2O-exch. $SO_3\underline{H}$); ^{13}C NMR (100 MHz, acetone-d_6), 13.7 ($\underline{C}^{11}$), 17.6 ($\underline{C}^2$), 21.5 ($\underline{C}^5$), 22.4 ($\underline{C}^{10}$), 26.0 ($\underline{C}^6$), 28.7 ($\underline{C}^7$), 28.9 ($\underline{C}^8$), 31.6 ($\underline{C}^9$), 47.9 ($\underline{C}^3$), 56.7 ($\underline{C}^1$), 58.7 ($\underline{C}^4$).

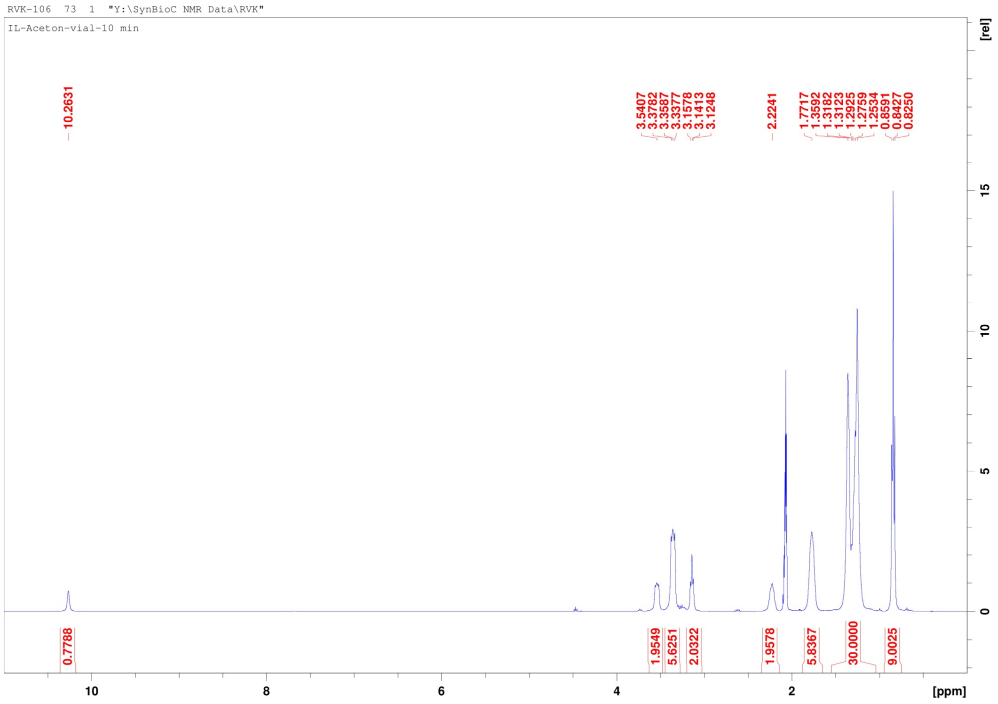

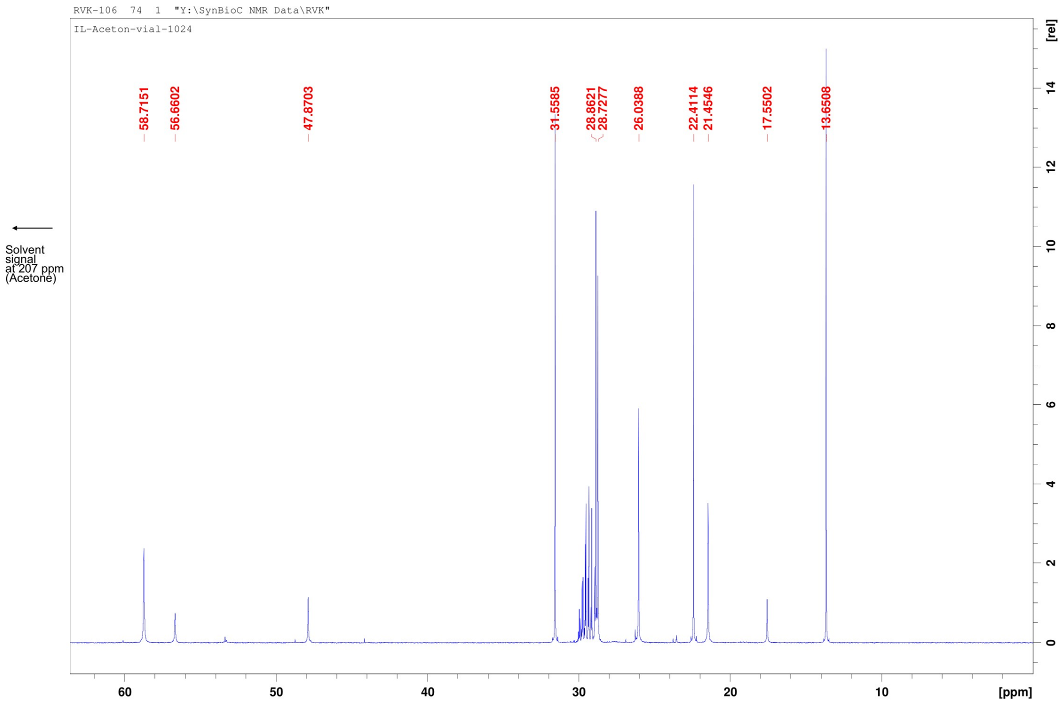

2. 2,4,6-Tribromophenol

2,4,6-Tribromophenol: ^{1}H NMR (400 MHz, $CDCl_3$): δ=5.85 (s, 1H, O<u>H</u>), 7.59 (s, 2H, $C^{3,5}$<u>H</u>); ^{13}C NMR (100 MHz, $CDCl_3$): δ=110(<u>C</u>^{2,6}Br), 112 (<u>C</u>^{4}Br), 134 ($C^{3,5}$H), 149 (C^{1}OH). This is in accordance with the online available spectra [1].

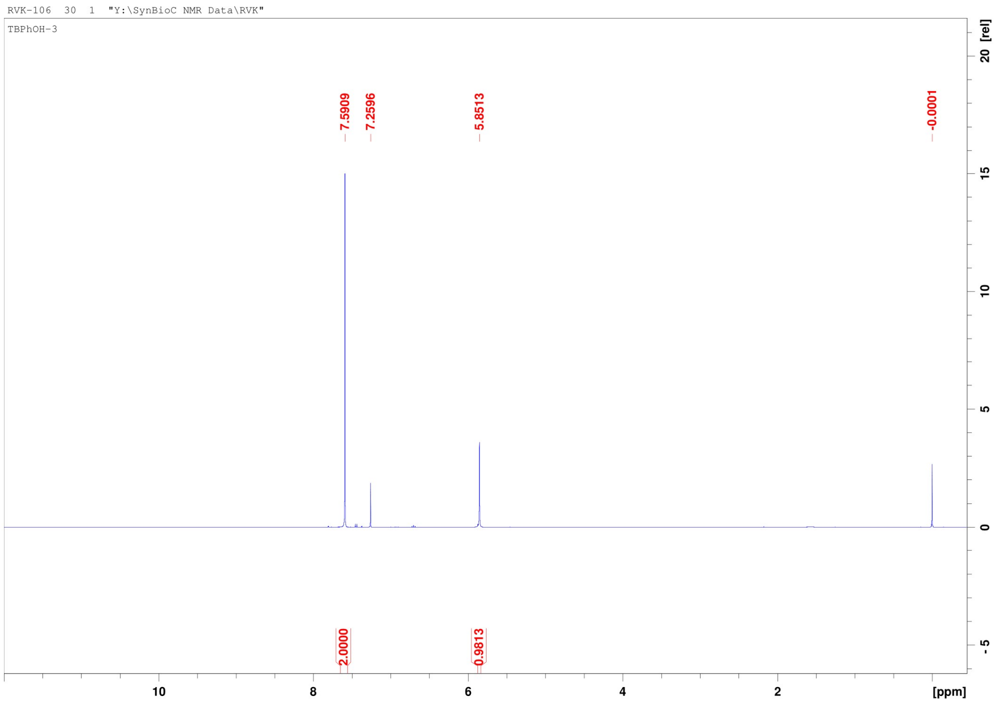

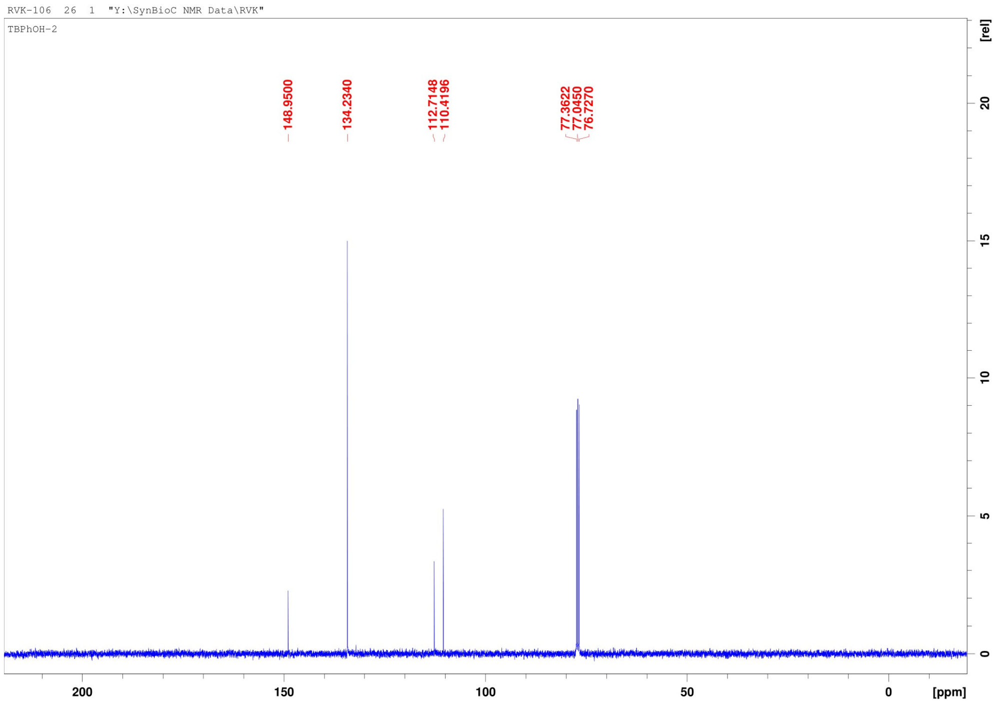
RVK-106 26 1 "Y:\SynBioC NMR Data\RVK"
TBPhOH-2
148.9500
134.2340
112.7148
110.4196
77.3622
77.0450
76.7270
[rel]
20
15
10
5
0
200
150
100
50
0
[ppm]

26

```
Data File Z:\2017\170607\ASCENTIS 20170524 2017-06-07 10-37-36\RVK-94-NO.D
Sample Name: RVK-94-NO

    =====================================================================
    Acq. Operator   : JB                              Seq. Line :   6
    Acq. Instrument : Instrument 1                     Location : Vial 21
    Injection Date  : 7/06/2017 11:16:35                    Inj :   1
                                                     Inj Volume : 2.0 µl
    Acq. Method     : D:\LCMS\DATA\170607\ASCENTIS 20170524 2017-06-07 10-37-36\NEG ASCENTIS
                      CH3CN
    Last changed    : 12/01/2015 9:22:18 by JB
    Analysis Method : Z:\190330\ASCENTIS 20190321 2019-03-30 15-07-47\ASCENTIS CH3CN LCMS POLAIR
                      DPN2 2.M
    Last changed    : 30/03/2019 15:27:36 by SD
    Method Info     : Ascentis column method for Synthesis samples
```

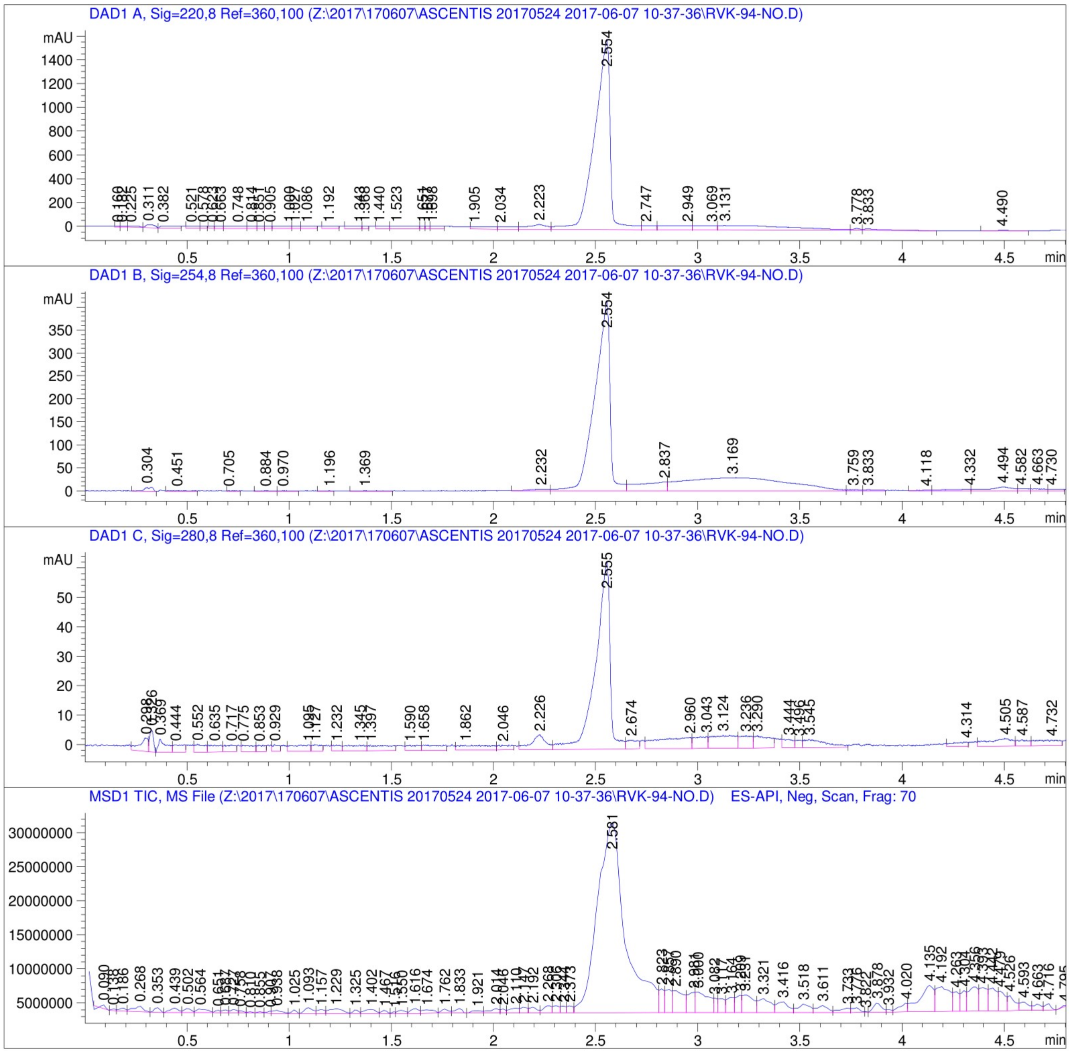

Instrument 1 29/05/2019 18:38:48 Page 1 of 5

27

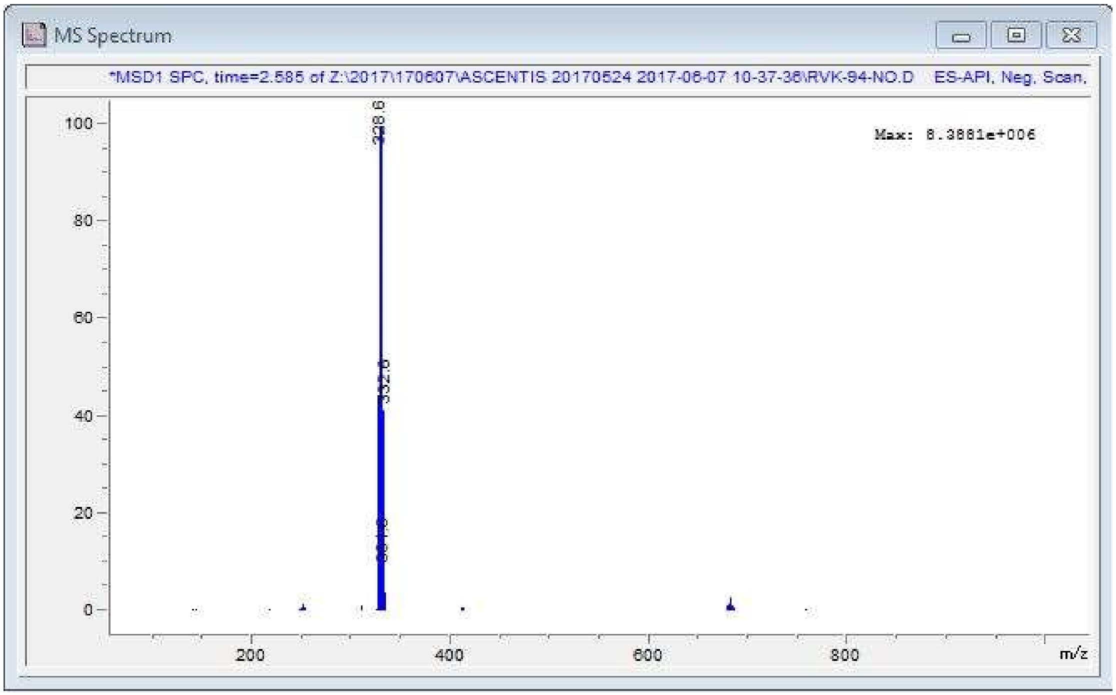

3. 2,2′,6,6′-Tetrabromobisphenol A

2,2′,6,6′-Tetrabromobisphenol A: ^{1}H NMR (400 MHz, MeOD-d_4): δ=1.61 (s, 6H; CH_3), 4.91 (s, D_2O-exch., OH), 7.30 (s, 4H, CH); ^{13}C NMR (100 MHz, MeOD-d_4): δ=30 (CH_3)$_2$, 41 (C_{quat}), 111 (CBr)$_4$, 130 (CH)$_4$, 144 (CC)$_2$, 149 (CO)$_2$ This is in accordance with the online available spectra [2].

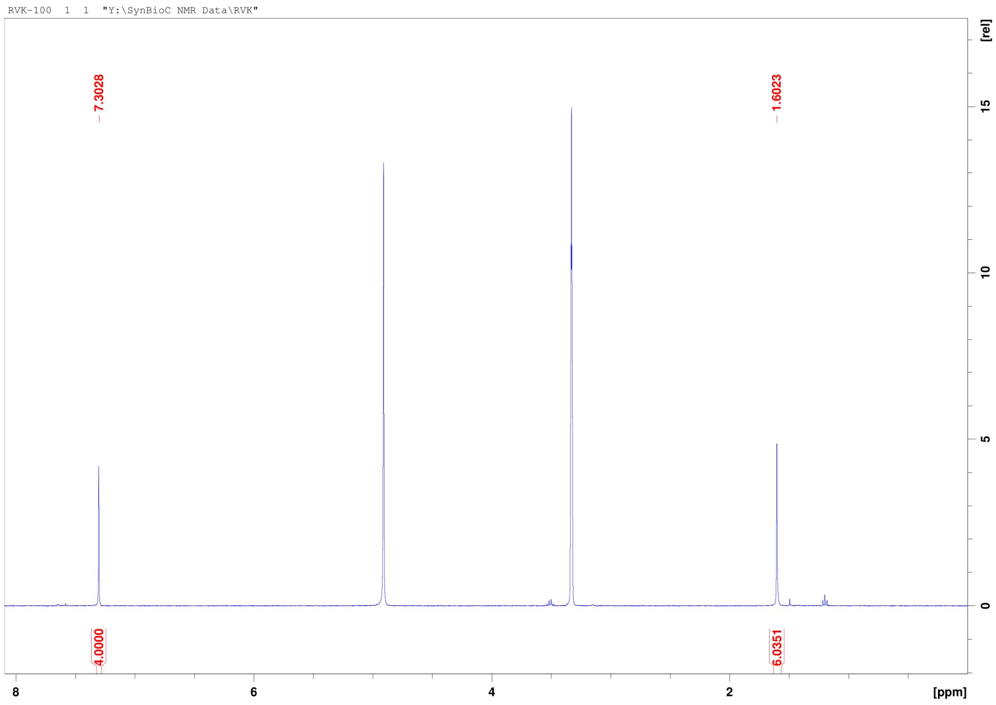
RVK-100 1 1 "Y:\SynBioC NMR Data\RVK"
7.3028
1.6023
4.0000
6.0351
[rel]
[ppm]

33

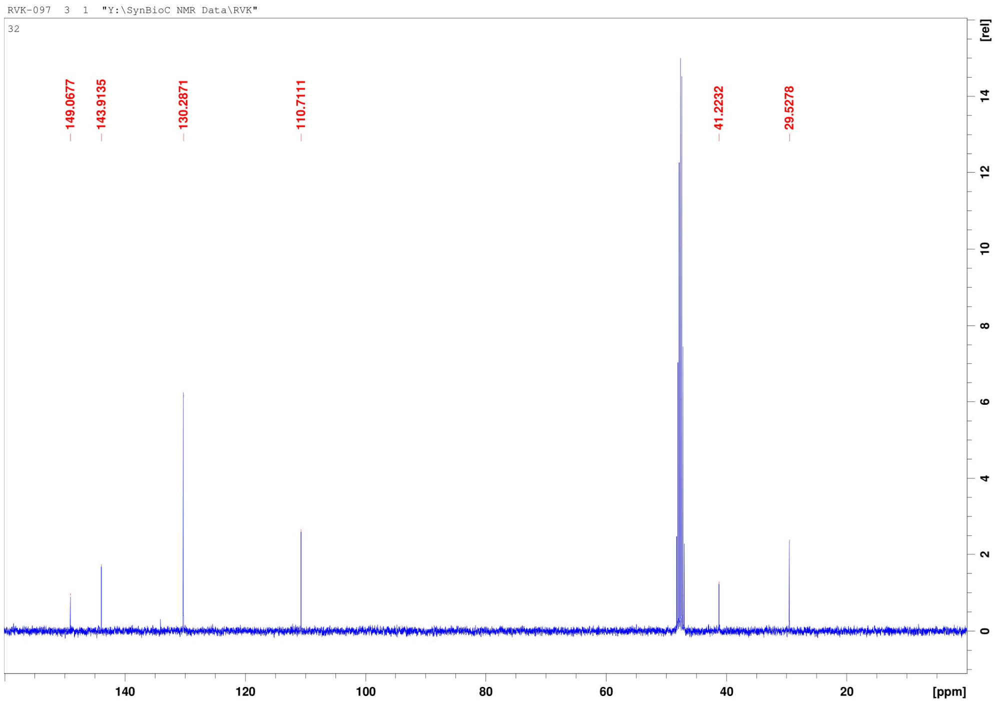
RVK-097 3 1 "Y:\SynBioC NMR Data\RVK"
32
149.0677
143.9135
130.2871
110.7111
41.2232
29.5278
[rel]
[ppm]

34

```
Data File Z:\2017\170612\ASCENTIS 20170524 2017-06-12 14-58-11\RVK-100-NO.D
Sample Name: RVK-100-NO

    =====================================================================
    Acq. Operator    : YM                            Seq. Line :   2
    Acq. Instrument  : Instrument 1                   Location : Vial 43
    Injection Date   : 12/06/2017 15:06:39                 Inj :   1
                                                    Inj Volume : 2.0 µl
    Acq. Method      : D:\LCMS\DATA\170612\ASCENTIS 20170524 2017-06-12 14-58-11\NEG ASCENTIS
                       CH3CN
    Last changed     : 12/01/2015 9:22:18 by JB
    Analysis Method  : Z:\190330\ASCENTIS 20190321 2019-03-30 15-07-47\ASCENTIS CH3CN LCMS POLAIR
                       DPN2 2.M
    Last changed     : 28/05/2019 14:53:15
                       (modified after loading)
    Method Info      : Ascentis column method for Synthesis samples
```

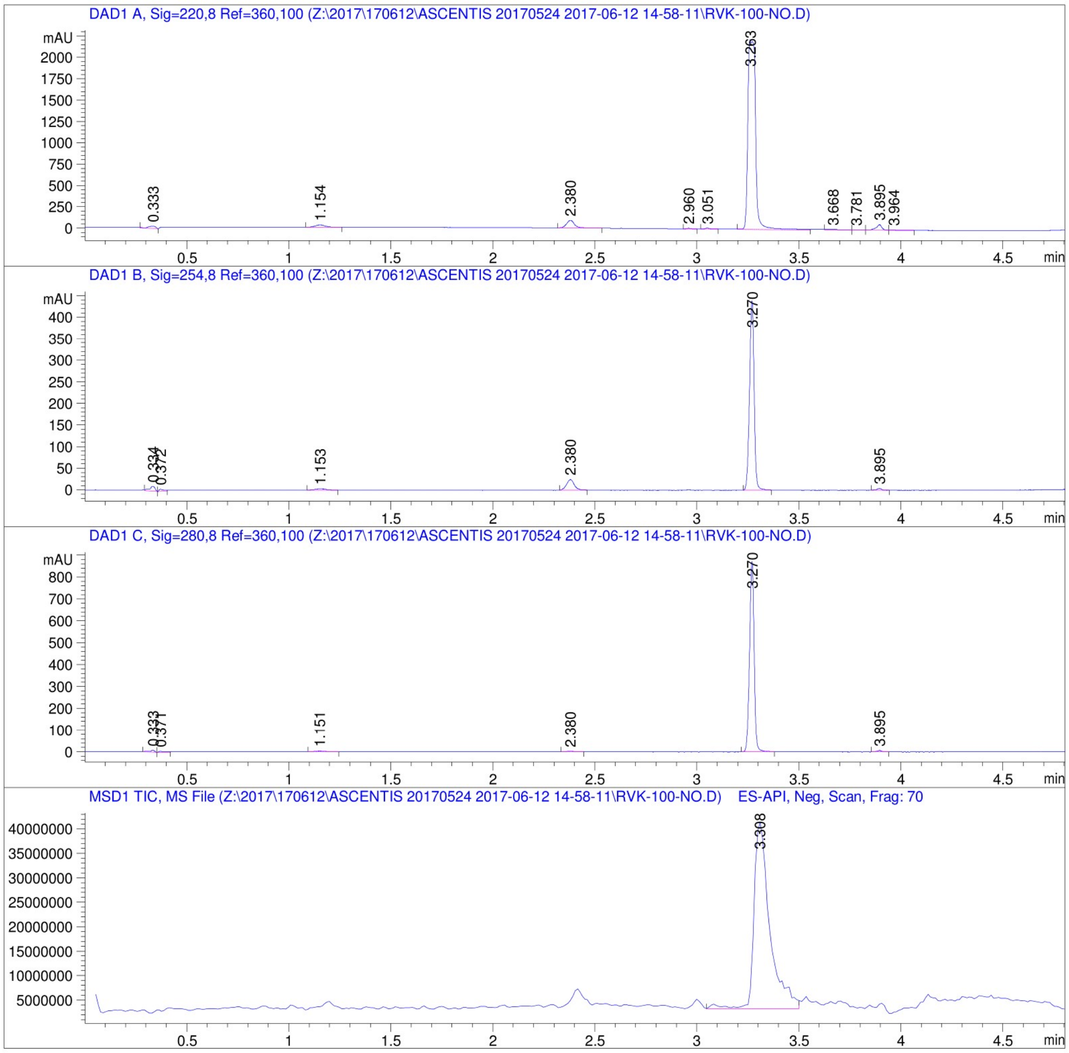

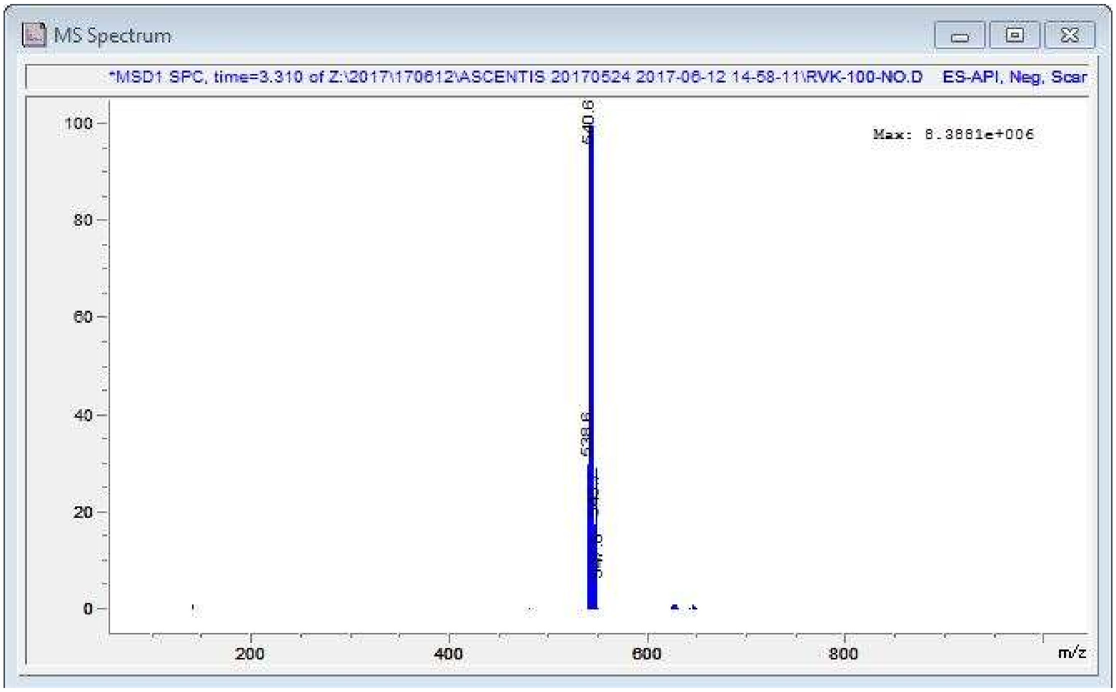

4. 1,2,5,6,9,10-Hexabromocyclododecane

Due to the multitude of chiral positions this product could not be analysed with NMR. Since the industrial application of hexabromocyclododecane, being an additive flame retardant, relies solely on the amount of bromine present and not on the enantiomeric or diastereomeric structure, checking the mass on LC-MS to detect possible underbromination, producing the unsaturated tetrabrominated product, or overbromination, producing the hepta- or octabrominated product was deemed sufficient. It can be seen on the LC-MS that there are multiple diastereomers, but no products with a different mass.

```
Data File Z:\2018\180702\ASCENTIS 20180417 2018-07-02 13-32-24\RVK-215-1MIN.D
Sample Name: RVK-215-1min

    =====================================================================
    Acq. Operator   : LDE                            Seq. Line :   1
    Acq. Instrument : Instrument 1                    Location : Vial 32
    Injection Date  : 2/07/2018 13:34:52                   Inj :   1
                                                    Inj Volume : 2.0 µl
    Acq. Method     : D:\LCMS\DATA\180702\ASCENTIS 20180417 2018-07-02 13-32-24\NEG ASCENTIS
                      CH3CN
    Last changed    : 12/01/2015 9:22:18 by JB
    Analysis Method : Z:\190330\ASCENTIS 20190321 2019-03-30 15-07-47\ASCENTIS CH3CN LCMS POLAIR
                      DPN2 2.M
    Last changed    : 30/03/2019 15:27:36 by SD
    Method Info     : Ascentis column method for Synthesis samples
```

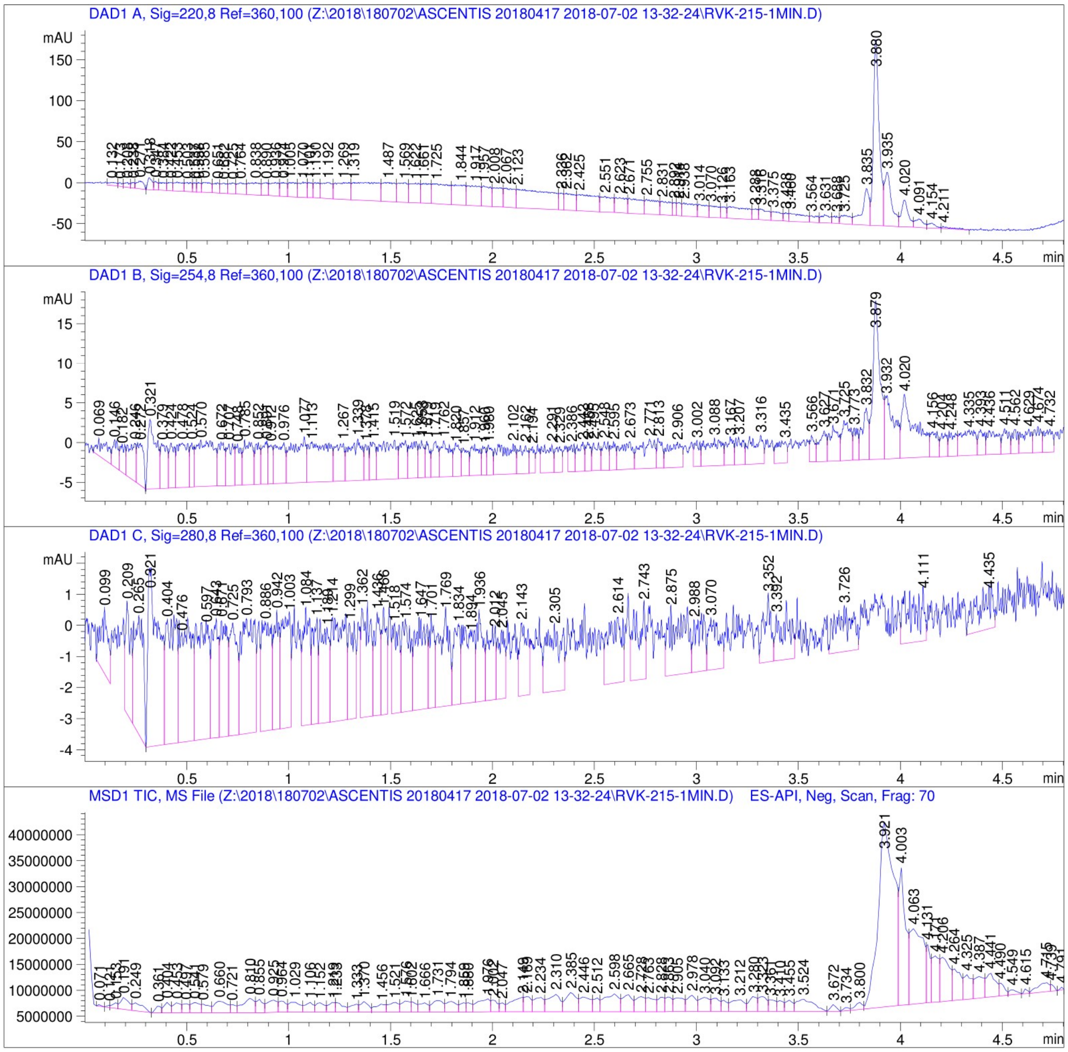


```
Instrument 1 24/05/2019 9:50:58                                           Page    1 of 7
```

The MS-spectrum at 3.921 minutes is given below:

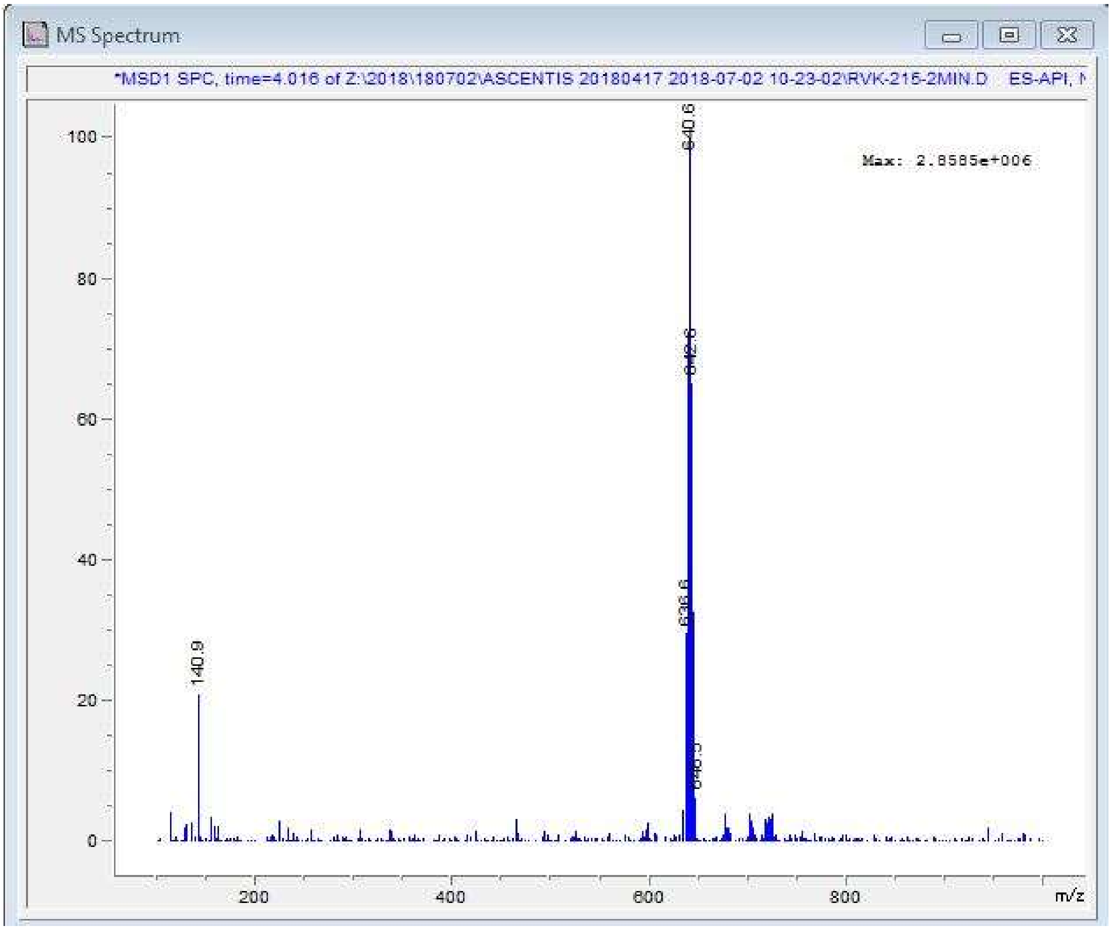

5. Eosin Y

The ^{1}H-NMR spectrum of eosin Y is difficult to interpret, because it appears as two tautomeric forms. The difference between these two can be seen in the 3 position, and the partial disappearance of the C^{5}OH-proton, and a slight shift of the 12a-b position The ratio between the two tautomers is approximately 7/3. This ratio is not fixed, and the equilibrium can be shifted by addition of D_2O. The effects of this tautomerism are too small to notice in the ^{13}C-NMR spectrum.

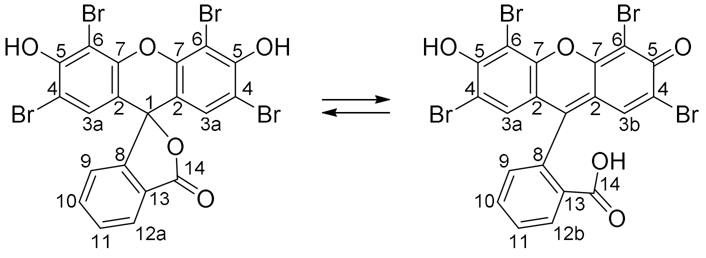

Eosin Y: ^{1}H NMR (400 MHz, DMSO-d$_6$): δ=6.91 (s, 1.4H, $C^{3a}\underline{H}$), 7.11 (s, 0.6H, C^{3b}H), 7.48 (d, J=7Hz, 1H, $C^{9}\underline{H}$), 7.77 (t, J=7Hz, 1H, $C^{11}\underline{H}$), 7.84 (t, J=7Hz, 1H, $C^{10}\underline{H}$), 8.03 (d, J=7Hz, 0.7H, $C^{12a}\underline{H}$), 8.18 (d, J=7Hz, 0.3H, $C^{12b}\underline{H}$), 10.82 (br. s, 1.6H, $C^{5}O\underline{H}$); ^{13}C NMR (100 MHz, DMSO-d$_6$): δ=81 ($\underline{C}^{1}$), 101 ($\underline{C}^{4}$), 108 ($\underline{C}^{6}$), 113 ($\underline{C}^{2}$); 124.7 ($\underline{C}^{9}$), 125.7 ($\underline{C}^{12}$), 126.2 ($\underline{C}^{11}$), 130 ($\underline{C}^{3}$), 131 ($\underline{C}^{13}$), 137 ($\underline{C}^{10}$), 148 ($\underline{C}^{5}$), 151 ($\underline{C}^{8}$), 154 ($\underline{C}^{7}$), 168 ($\underline{C}^{14}$). This is in accordance with the online available spectra [4].

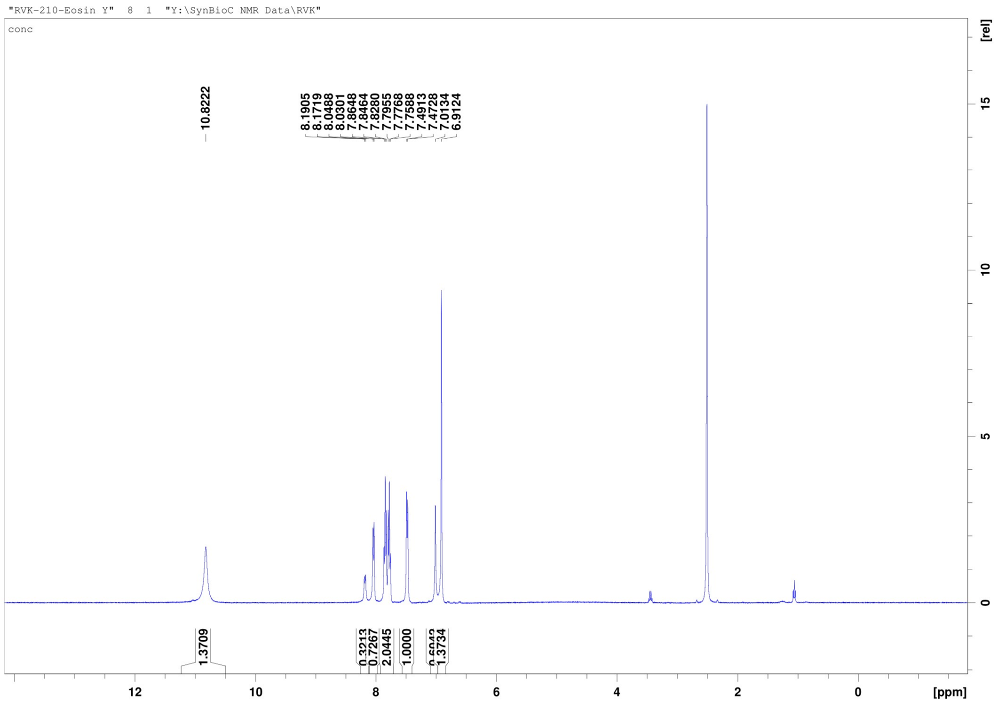
"RVK-210-Eosin Y" 8 1 "Y:\SynBioC NMR Data\RVK"
conc
10.8222
8.1905
8.1719
8.0488
8.0301
7.8648
7.8464
7.8280
7.7955
7.7768
7.7588
7.4913
7.4728
7.0134
6.9124
1.3709
0.3213
0.7267
2.0445
1.0000
1.3734
[rel]
[ppm]

69

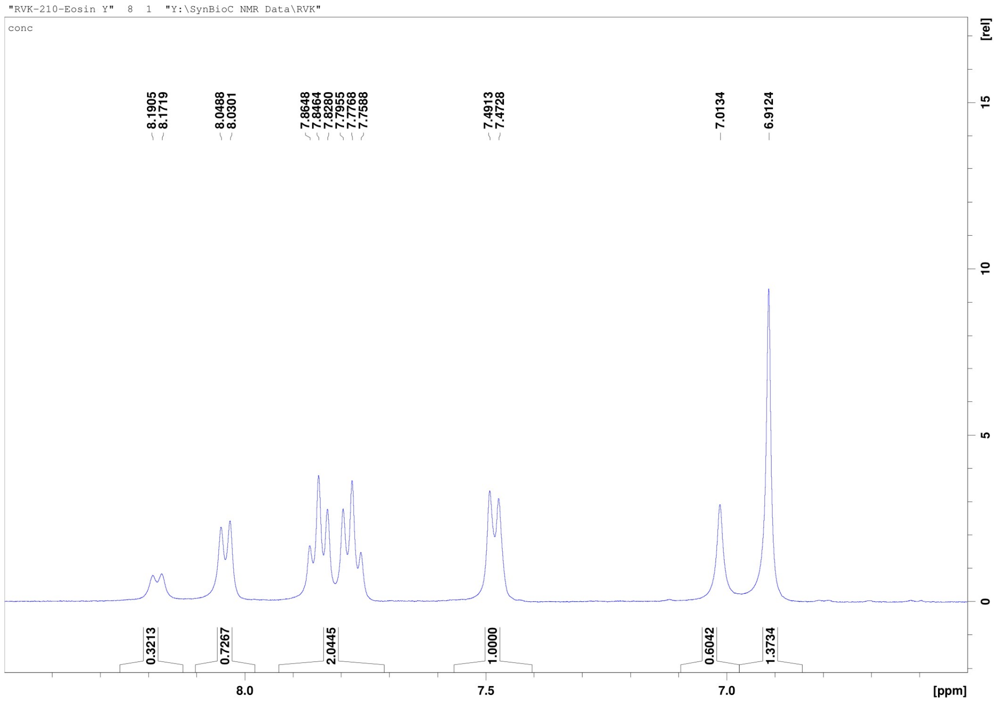
"RVK-210-Eosin Y" 8 1 "Y:\SynBioC NMR Data\RVK"
conc
8.1905
8.1719
8.0488
8.0301
7.8648
7.8464
7.8280
7.7955
7.7768
7.7588
7.4913
7.4728
7.0134
6.9124
0.3213
0.7267
2.0445
1.0000
0.6042
1.3734
[rel]
[ppm]

70

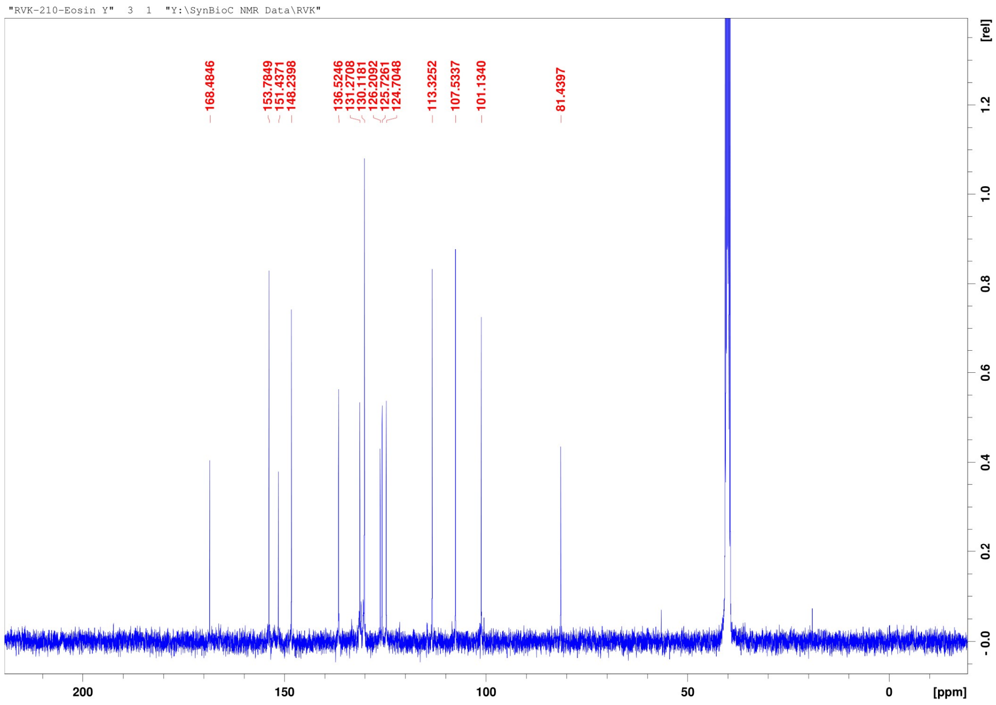
"RVK-210-Eosin Y" 3 1 "Y:\SynBioC NMR Data\RVK"
168.4846
153.7849
151.4371
148.2398
136.5246
131.2708
130.1181
126.2092
125.7261
124.7048
113.3252
107.5337
101.1340
81.4397
[rel]
1.2
1.0
0.8
0.6
0.4
0.2
0.0
200
150
100
50
0
[ppm]

71

Data File Z:\190528\ASCENTIS 20190425 2019-05-28 15-58-27\RVK-EOSIN Y.D
Sample Name: RVK-Eosin Y

===

```
Acq. Operator    : SD                              Seq. Line :  15
Acq. Instrument  : Instrument 1                     Location : Vial 96
Injection Date   : 28/05/2019 17:51:15                   Inj :   1
                                                  Inj Volume : 2.0 µl
Acq. Method      : D:\LCMS\DATA\190528\ASCENTIS 20190425 2019-05-28 15-58-27\NEG ASCENTIS
                   CH3CN
Last changed     : 28/12/2016 19:44:32 by YM
Analysis Method  : Z:\190330\ASCENTIS 20190321 2019-03-30 15-07-47\ASCENTIS CH3CN LCMS POLAIR
                   DPN2 2.M
Last changed     : 28/05/2019 14:53:15
                   (modified after loading)
Method Info      : Ascentis column method for Synthesis samples
```

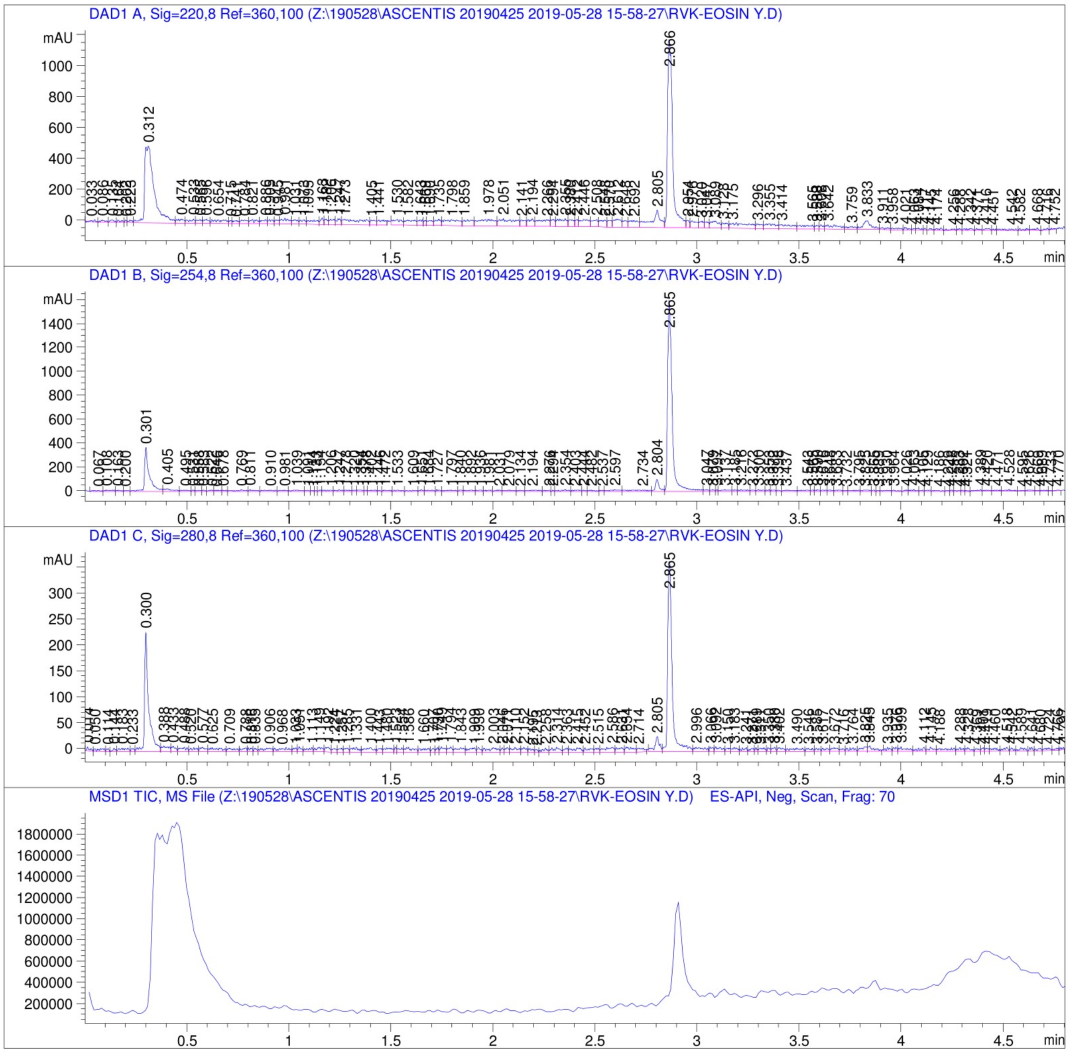

Instrument 1 29/05/2019 17:43:48 Page 1 of 8

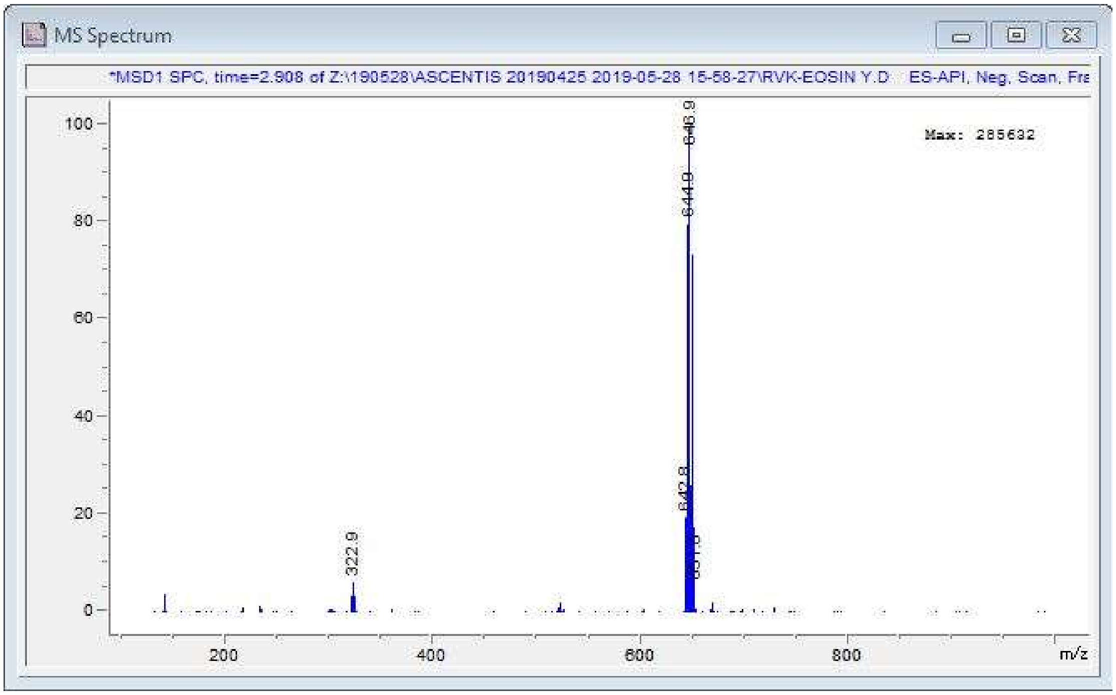

References

1. Sigma Aldrich spectral database 2,4,6-Tribromophenol. *Merck* https://www.sigmaaldrich.com/spectra/fnmr/FNMR001079.PDF
2. Sigma Aldrich spectral database 2,2',6,6'-Tetrabromobisphenol A *Merck* https://www.sigmaaldrich.com/spectra/fnmr/FNMR006985.PDF
3. SDBS Bromothymol Blue. *National Institute of Advanced Industrial Science and Technology (AIST)* https://sdbs.db.aist.go.jp/sdbs/cgi-bin/landingpage?sdbsno=1762
4. SDBS 2',4',5',7'-Tetrabromofluorescein. *National Institute of Advanced Industrial Science and Technology (AIST)* https://sdbs.db.aist.go.jp/sdbs/cgi-bin/landingpage?sdbsno=7510

© 2019 by the authors. Submitted for possible open access publication under the terms and conditions of the Creative Commons Attribution (CC BY) license (http://creativecommons.org/licenses/by/4.0/).
